# Evaluation of Mexican poverty reduction policies under the COVID-19 pandemic impacts

**DOI:** 10.3389/fpubh.2022.978991

**Published:** 2022-10-11

**Authors:** Gou Shuying, Liu Xuedong

**Affiliations:** ^1^Center for Latin American and Caribbean Studies, Southwest University of Science and Technology, Mianyang, China; ^2^School of Humanities and Social Sciences, University of Science and Technology of China, Hefei, China; ^3^School of Higher Studies Aragon, National Autonomous University of Mexico, Mexico City, Mexico

**Keywords:** extreme poverty, multidimensional perspective, poverty rate, vulnerable population, transfer income

## Abstract

From 2019 to 2020, the Mexican economy declined for two consecutive years, especially in the last one when it was hit by a decline of 8.4% before the COVID-19 pandemic impacts which was not only one of the worst in the OECD club, but also the deepest economic recession since 1932 in the national history. At the same time, both the number of people in poverty and poverty rate in 2020 have increased compared with those registered in 2018. Through the analysis, we can find that the current Mexican government has increased the intensity and scope of the implementation of social relief policies adhered to the principal of “for the good of all, first the poor (Por el bien de todos, Primero los pobres).” However, in the context of recession caused by the COVID-19, neither the general decrease in residents' income could be avoided, nor the number of people in poverty has been reduced. Besides, in accordance with the benefits obtained by the distinct household deciles based on the income and expenditure survey published by INEGI, it showed that the implementation of government relief measures has relatively reduced the support for the low-income people and further aggravated the deterioration of poverty due to its indifferent application with respect to high-income households and the low-income ones. Therefore, the deficiencies in the response implemented in the face of the epidemic, especially poverty alleviation actions and social relief policies, have further enhanced the poverty problem at least partially. In this sense, recover and improve the economic growth rate as soon as possible will not enough to reduce the poverty, and it should be accompanied by the necessary adjustments in the poverty alleviation measures and social relief policies, especially with a focalized approach inclined to the low-income segments of the population.

## Research background and literature review

Poverty is one of the most acute social problems in the design and application of social politics and its elimination has been considered as a great challenge faced by all countries in the world today which has attracted the attention of both the governments and all sectors of society.

The hypothesis of this study is that actual public policies applied by the government to alleviate the poverty problems until now should be adjusted with more focalization, besides the efforts necessary to overcome the crisis caused by the COVID-19 and regain the further economic growth rate.

As it can be observed that the analysis is carried out with a clear objective: current Mexican government has increased the public expenditure in the implementation public policies with the purpose to back up the families considered in poverty, holding the principle of taking care of the poor first; however, they cannot make up the losses caused by economic recession in face of the COVID-19. In this sense, there are more people dropped in poverty than before, since their personal income was lower than the poverty line in 2020, and/or some of them have been deprived at least one of the 6 social rights determined by the CONEVAL.

This job is structured in 3 sections after he presented research background and literature review. In the first part, it describes the evolution of the concept of poverty and its definition of poverty standards in Mexico; consequently, the COVID-19 pandemic's influence on Mexican poverty will be analyzed; finally, son conclusions.

The research on the problem of poverty has a history of more than 100 years, its understanding and acknowledgments have developed from the concept of absolute poverty to relative one; and its measurements, from single index to multi-dimensional approaches and from income poverty to ability poverty (1). In some cases, several scholars even associated it with the definition of middle class, thus proposing that there may be some population groups that are probable neither poor nor middle class (2, 3).

At the initial moment, most of the researches defined poverty from the perspective of maintaining people's basic living needs. Rowntree was the first scholar to put forward the concept of absolute poverty and considered the poverty as the population segment whose total income is not enough to afford the minimum necessities needed to maintain normal physical function, including food, clothes, housing and so on (4). On the basis of the minimum living expenditure or the poverty line, then he calculated the proportion of poor people according to this poverty line called poverty rate. Pete Alcock further deepened this concept, pointing out that the objective concept of absolute poverty was based on “subsistence” (5).

With the further understanding of poverty, the connotations of the poverty had gradually increased, which not only meant that the basic living needs cannot be satisfied, but also implicated that they suffered from relative exclusions and deprivations. Therefore, the theory of relative poverty was put forward. Fuchs Victor was the first to explicitly pointed out the concept of relative poverty. He suggested the relative poverty line as 50% of the median income of the national population and on the behave of it, proceeded to estimate the relative poor population in the United States. This method was used by many scholars later, and according to the World Bank standards, people whose incomes are < 1/3 of the average level can be regarded as relatively poor (5).

In 1970s and 1980s, Amartya Sen first mentioned the concept of “ability poverty.” He held in his book “Looking at Development with Freedom” that poverty is not only due to the low-income, but also means the deprivation of basic abilities (6). Sen's viewpoint was a milestone in the study of poverty theory, which extended its scope from a single economic factor to political, economic, cultural, and institutional aspects, and made people's cognition of the phenomenon of poverty extend from one-dimensional to multi-perspective and multi-dimensional.

Both the absolute and relative approaches, the poverty has been analyzed only from a single perspective, which often makes it difficult to comprehensively and truly reflect human development and poverty in other dimensions except income. Therefore, the concept of multidimensional poverty carried out by Sen has renewed the acknowledgments for this particular social phenomenon, so as to measure in which dimension(s) a person has been deprived of the freedom to develop his capacity, and then cause his life to fall into a situation in vulnerability or in poverty.

After the United Nations Development Programme (UNDP) released the Human Development Report 2010, the concept of multidimensional poverty began to spread and be accepted widely around the world. The United Nations Development Program, the World Bank and the Research Center for Poverty and Human Development (OPHI) of Oxford University are some of the most important multilateral institutions engaged in the study of multidimensional poverty. Among them, UNDP and OPHI published the Global Multidimensional Poverty Index (MPI) jointly replaced the Human Poverty Index (HPI) released by the United Nations Human Development Report, including 10 indicators in 3 dimensions of health, education and living standards (1). Meanwhile, the Economic Commission for Latin America (CEPAL) has published many reports on multidimensional poverty in Latin America and the Caribbean, and several countries in the region, such as Mexico (7) and Chile (8), have also formulated specific indicators for measuring multidimensional poverty according to their respective conditions.

## Evolution of the concept of poverty and its definition of poverty standards in Mexico

Since 2009, Mexico has initiated the studies to define the poverty and its measurement from two aspects: personal income of residents and six human social development indicators. Every 2 years, it regularly publishes reports on poverty in local states and the whole country; since 2010, the relevant data has been further extended to the municipal level.

### Institutional setting and the measurement of poverty

The phenomena of poverty in Mexico have been understood and managed as the only economic concept traditionally for a long time, as in other countries of the world. In this sense the personal disposable income is almost the unique indicator to measure the poverty situation of the household. Therefore, this one-dimensional view based on the minimum income or consumption amount needed by a person to afford the basic basket of products as the poverty line, and those people whose income is below this minimum threshold should be considered to be in the poverty.

On 20 January 2004, Mexico promulgated the Basic Law on Social Development, which established the National Commission for the Evaluation of Social Development Policies (Consejo Nacional de Evaluación de la Pol í tica de Desarrollo Social, CONEVAL). The Agency is an independent technical unit under the federal government, which specializes in the evaluation of public social development policies and the determination, identification and measurement of poverty. At the same time, the Basic Law of Social Development also laid the foundation for the multidimensional definition and measurement of poverty. It pointed out that poverty should not only consider the economic welfare reflected by personal income, but also include six other indicators related to social rights.

Since 2008, CONEVAL has officially launched the multidimensional poverty measurement in the country at the national, the state and municipal levels, respectively. In 2020 when the latest version of the poverty report was published, it can be seen that with a periodicity of every 2 years during the last 12 years the information has been actualized on the basis of the data released by the National Institute of Informatics and Statistics (INEGI in abbreviation and with an interval of 2 years too in the release) in the National Survey of Household Income and Expenditure (Encuesta Nacional de Ingreso y Gasto de los Hogares, or ENGIH in abbreviation). During this period, although several adjustments have been made around poverty indicators and poverty connotations in order to improve the measurement of the poverty situation in Mexico, the comparability of data in these different reports has been basically maintained, because the original data used to calculate all of the detailing indicators related to every aspect of poverty, including historical poverty measurement data at the national, the state and the municipal levels, the deficiency of the 6 social freedom, are all from the same source.

(1). The identification and determination of social indicators to measure the poverty in the multidimensional approach.

In order to fully implement the social rights mentioned in article 36 of the Basic Law on Social Development, CONEVAL has designed six specific social rights indicators, in addition to personal income in the measurement of the poverty in the multidimensional approach, specifically they are: education backward, health care services, social security, quality and size of housing, basic housing services and access to food. Therefore, CONEVAL will examine the poor population from two aspects of the household and its members. First, the economic welfare is measured by personal income to determine the number of people in the poverty situation. Specifically, this group of residents refers to those people whose personal income is insufficient to purchase all kinds of goods and services stipulated in the basic products basket or the income poverty line. Among them, this segment of population or households can be cataloged into moderate and extreme ones according to the income amount. Both of the two groups of population or households whose personal income is lower than the amount fixed by the poverty line and the differences between them is that the former refers to the segment whose personal income can afford the basic foods basket to meet their daily living needs; and meanwhile the latter, to those who can't catch up the basic foods basket even if they spend all their income on the same basket.

Second, in terms of social rights, anyone who suffers the shortage of the six social right indicators will be defined as people in poverty due to the shortage of social rights. With the increase of lacking number of the social rights, it means that the degree of poverty rises. When the number of lacks of social rights sums to three or more, they will be defined as multidimensional extreme poverty groups.

(2). Combining income indicators with social rights to measure poverty.

**Figure 1:**
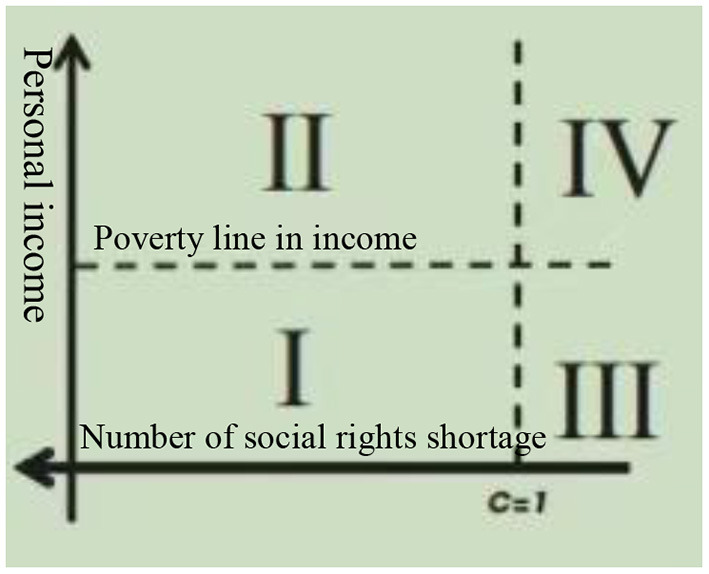
Classification of population under multi-dimensional indicators approach. Source: Consejo Nacional de Evaluación de la Política de Desarrollo Social (7), Page 37.

Through the combination both the income and the deprivation of social rights, it can define and measure the population in poverty from two aspects, so that the multidimensional poverty can be quantified more accurately. In Mexico, the CONEVAL uses theses judgments to classify the population into four groups in accordance with the income levels and the deprivation of social rights and shown in Figure 1.

In Figure 1, the vertical axis represents the income indicator, on basis of it, the total national population or household could be cataloged into two groups: one in situation in poverty and another in no poverty according to the income poverty line (or welfare standard line) calculated and actualized every month by CONEVAL.

On the other hand, the horizontal axis indicates the lack of the social rights amounts, and in the origin of coordinates the shortage reaches its the maximum value of 6, representing those residents who lack all of the social rights; In the right part of C=1, it means that residents do not suffer any lack of social rights. Those residents whose shortage of social rights situate between 1 and 6 belong to those in poverty due to the lack of social rights. In addition, according to CONEVAL's calculation method, people with a lack of rights of 3 or more are classified in the extremely multidimensional poverty.

Therefore, every resident can be classified into one of the four quadrants in Figure 1 according to the index of personal income and lack of social rights:

- Population in multidimensional Poverty. Refers to the population whose income is below the poverty line and lacks at least one social right, that is, in Area I in Figure 1, which is also the basis for calculating the poverty rate in Mexico.
- Population in vulnerability due to the shortage of social rights. Refers to the group of population who lacks at least one social right but whose income is equal to or higher than the poverty line, correspondence to the Area II of Figure 1.
- Population in vulnerability due to income. This group does not suffer from the lack of social rights, but whose income is below the poverty line, and it is in Zone III in Figure 1.

It should be pointed out that people in vulnerability due to income or social rights only do not belong to poverty according to the current standards set by Mexico, and some of them are in the buffer stage from poverty to middle class, while others belong to middle class (9). With the improvement of the economic situation, this group of people will gradually get rid of vulnerability and enter the non-poor and non-vulnerable classes; however, in the case of severe economic recession, they are very likely to fall into poverty groups.

- Population neither in poverty nor in vulnerability. This group refers to the population whose income is equal to or higher than the poverty line and at the same time they do not suffer from any shortage of social rights, that is correspondent to the Area IV in Figure 1. They could belong to either the middle class or the rich group, and have strong resistance to unfavorable environment. Even in times of economic crisis, it is difficult to live in poverty or vulnerability.

**Figure 2:**
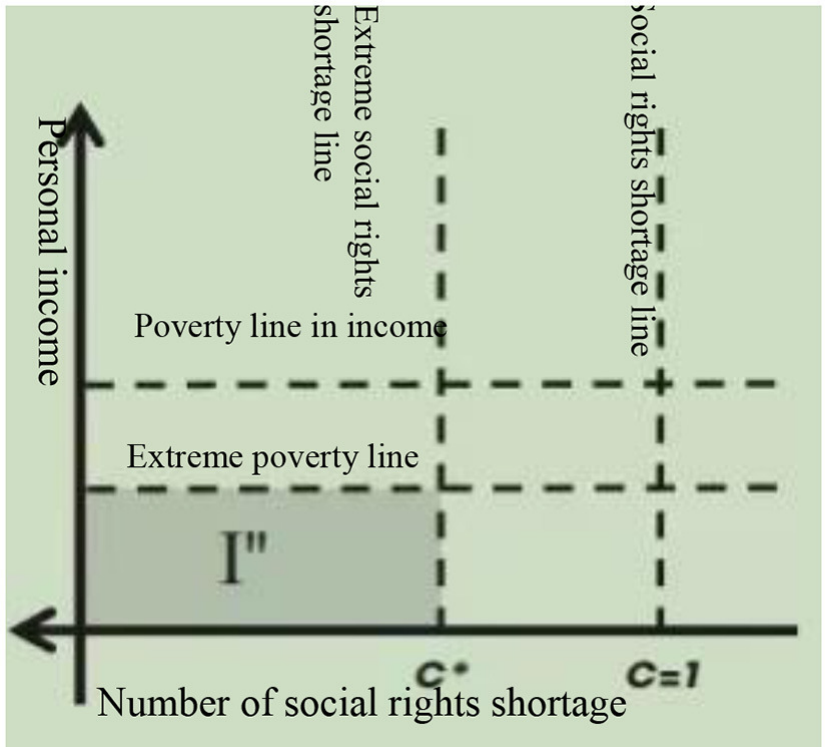
Extremely multidimensional poverty. Source: Consejo Nacional de Evaluación de la Política de Desarrollo Social (7) Page 38.

As mentioned before, the poverty could be cataloged into extreme poverty and moderate poverty, and each of them also could be distinguished due to income and due to shortage of social rights. For example, if the personal income is lower than the extreme poverty line and the number in the shortage of social rights is from 3 to 6, that is, 3 ≤ the number of lack of social rights ≤ 6, the residents would be included in Area II”, as it shows in Figure 2, they not only earn less than the cost of the basic foods basket they need, but also lack at least three of the six social rights.

### Contributions to improve the poverty measurement by Mexico

From 2008, the multidimensional poverty approach carried out for Mexico particularly through the CONEVAL definitely has enriched the acknowledgments and the measurements about the poverty; and at the same time, all of these efforts related to design, implementation, monitoring and evaluation of social policies have been shared and interchanged with nearly 20 countries from Asia, Europe, Africa and Latin America including China. Also, the CONEVAL actively carries out cooperation and international dialogue on poverty reduction issues with United Nations agencies, such as the Food and Agriculture Organization of the United Nations, the Economic Commission for Latin America and the Caribbean, and other international organizations such as the United Nations Development Programme, the World Bank and the Inter-American Development Bank (7), concentrated in the following aspects:

(1) Integrate the multidimensional poverty approach and the poverty measurements into the legal framework through the institutionalization.

The criteria and poverty lines established for each dimension within the multidimensional poverty approach have been set in accordance with the Mexican Constitution and the main laws and regulations in the social field, without doubt also offer a guarantee to carry out the basic principles of public social development policy and the basic economic benefits and social rights for all citizens. Meanwhile the most important of the framework consists in an independent technical unit under the federal government, namely the National Social Development Policy Evaluation Committee (CONEVAL), which specializes in the evaluation of public social development policies and the definition, identification and measurement of poverty.

All of the adequately arranged institutional frameworks related to the multidimensional approach in Mexico have gradually formalized the methodologies in the poverty studying and in the processing of the collected information according to each of the indicators previously established. Since CONEVAL officially launched the multidimensional poverty measurement in 2008, the poverty reports released in every 2 years from 2008 to 2020, have provided an important basis and reference in the design, implementation and evaluation of social public policies for all of the government departments involved in the poverty reduction issues and in the research for academic institutions.

(2) Adopt multidimensional methods to accurately identify the poor population.

In order to fully implement the social rights mentioned in Article 36 of the Basic Law on Social Development, CONEVAL defines and measures the number of people in poverty in terms of the personal income and the number of deprived social rights (six indicators). This multi-dimensional definition and measurement method of poverty is conducive to accurately identifying which situation of poverty that the particular segment of households can be cataloged, as well as the manifestations and degrees of poverty.

(3) Poverty measurement results have become an important basis for the formulation of public social policies in confrontation of the poverty reduction.

The poverty level report published regularly by CONEVAL every 2 years has gradually become an important basis for the formulation, implementation and monitoring of public social policies to improve the poverty reduction in Mexico.

Concretely, in the National Development Plan 2013–2018, it is clear that its main goal is to ensure the effective exercise of social rights by all people.

In the Sectoral Plan for Social Development 2013–2018, some of the indicators identified in the CONEVAL report are also used in the formulation of policies related to social development, and in the assessments of the detailed impacts of sectoral development policies and the progress they have made in eradicating poverty, improving living conditions and reducing the vulnerability.

Besides, the information released by the CONEVAL in its respective reports has provided all the authorities in the state and municipal levels related to the poverty through the signature of different cooperation agreements between the institution and the local governments in order to conduct poverty reduction evaluation and contribute to the state and the municipal poverty diagnosis.

Finally, CONEVAL's measurement and analysis of poverty and its assessment of public social policies serve as the basis for setting priorities in the federal government's budget and fiscal policies.

In fact, in basis of the diagnosis in relation to the extremely multidimensional poverty published by the CONEVAL, in January 2013, Mexico government issued the decree of “National Anti-Hunger Campaign;” in the same direction, the federal government initiated the creation of the Universal Pension Act (Ley de Pensión Universal) and the Unemployment Insurance Act (Ley de del Seguro de Desempleo), in accordance with the extent of the lack of social security revealed in the poverty report in 2012 (7, 10).

## COVID-19 pandemic's influence on mexican poverty

According to the results released by the National Survey of Household Income and Expenditure (ENGIH) in 2020 (11), the National Social Development Policy Evaluation Committee of Mexico published the results of poverty situation in 2020 (12) in early August 2021 (12). It not only shows the change of poverty rate calculated in accordance with the income level, but also related to the lack of six social rights. As this is the first poverty report since the current government (2018–2024) took office, it is certainly an important part of evaluating the administration performance. Therefore, for President Lopez, who has held the principle of “Por el bien de todos, primero los pobres,“ the report could be particularly important in this field.

### Brief introduction of poverty in 2018–2020

On the basis of the multidimensional approach in poverty measure described earlier, the number of people in poverty increased from 51.9 million to 55.7 million in the 2 years from 2018 to 2020 in Mexico, with a cumulative increase of 3.8 million. At the same time, the proportion of those in poverty in the total population also increased from 41.9 to 43.9%, an increase of 2 percentage points. In addition, from the perspective in the evaluation of the six social rights indicators, three of them, education, health care services and access to food, both in absolute and relative terms, showed a decline; meanwhile, the other three indicators, namely social security, housing quality and area, and basic housing services, have all improvement.

Among the 3 social rights with decrease, the health care services had the worst performance, because the number of residents who lack this service has increased greatly, from 20.1 million in 2018 to 35.7 million in 2020, and their proportion in the total population of Mexico has increased from 16.2% in 2018 to 28.2% in 2020. In the aspect of the educational services ~900 thousand personas registered the backward; and 1.1 million had the difficulties to get the access to nutritious and quality foods during the same 2 years.

According to the analysis, the sudden propagation of COVID-19 pandemic and the resulting economic recession are undoubtedly the main factors for the deterioration of the local poverty situation, which not only leads to the increase of the number of people in poverty, but also makes people's demand for medical services greatly increased due to the emergence of the epidemic, and the original infrastructure in this sector has been saturated in large quantities, and resulted in that the demand seriously exceeded the supply. But at the same time, it should be pointed out that this may only be one aspect of the problem. On the other hand, shortly after the current government took office, because it began to replace the Seguro Popular (SP) implemented many years ago by the Instituto Nacional de Salud para el Bienestar (INSABI), and the budget was cut down, the coverage capacity services were relatively reduced (13), resulting in several serious supply defects. In terms of amounts of person, that is a reduction of 20.4% in 2021 in comparison to the assigned in 2019. Therefore, even without the outbreak of COVID-19 pandemic, it would be very likely that there should be an increase in the number of people facing the shortage in the right to access the health care services; the sudden sanitary emergencies due to COVID-19 has led to further deteriorate the problem of health care services.

### Changes in the respective categories of poverty

#### Extreme poverty

**Figure 3:**
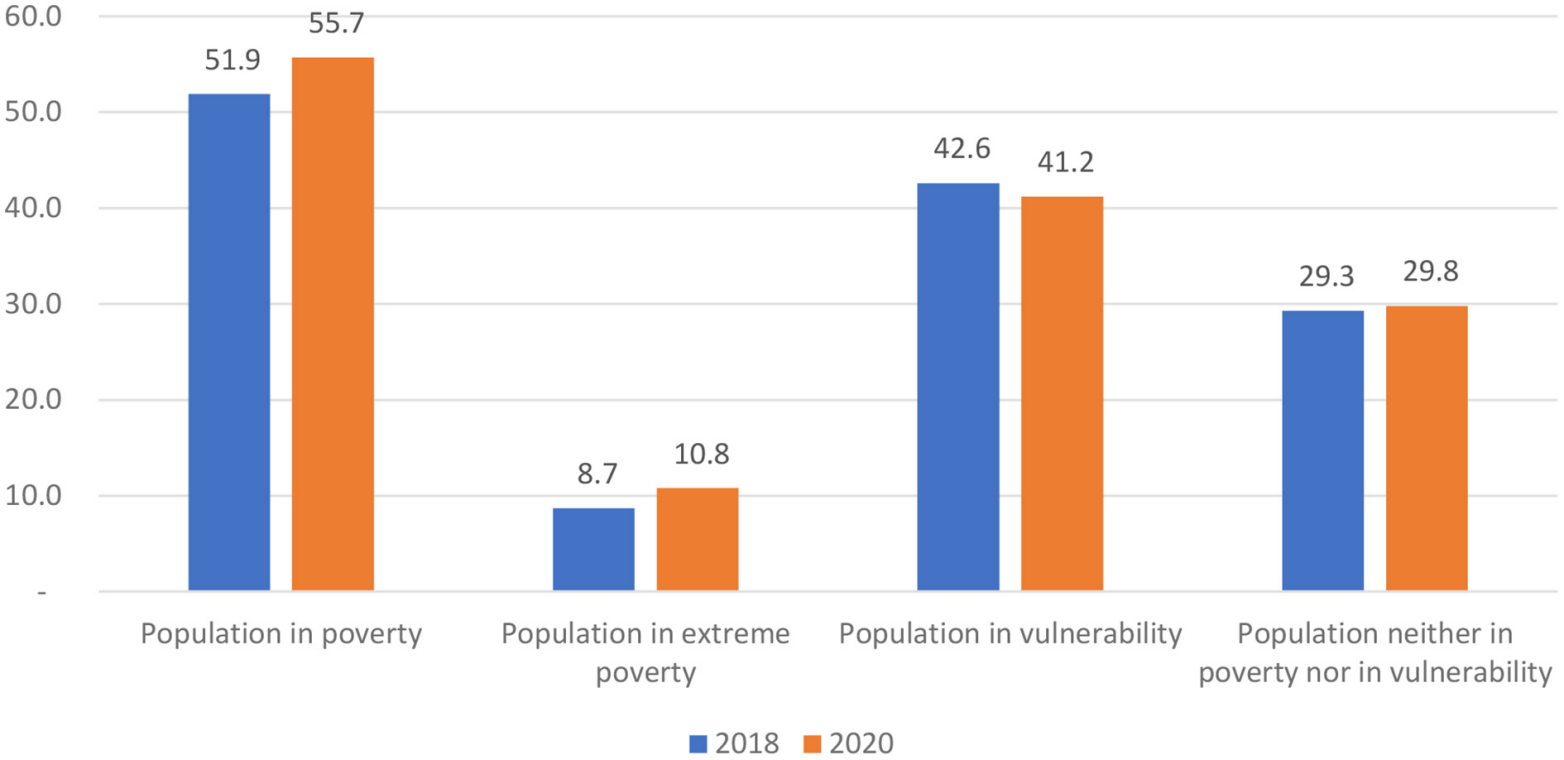
Changes in poverty categories in Mexico, 2018–2020 (million). Source: Based on data published by the Mexican National Commission for the Evaluation of Social Development Policies.

According to the data published in the report, among the total increase of 3.8 million people in poverty, the extremely poor accounted for more than half, with a net increase of 2.1 million who represented a 55.3%; in the absolute terms, the population in extreme poverty raised from 8.7 million to 10.8 million, and their proportion in the total Mexicans also ascended from 7.0 to 8.5% (as shown in Figure 3). At the same time, the population in moderate poverty, that is, the residents whose personal income exceeded the minimum welfare line but was lower than the standard welfare line, has increased from 43.2 million to 44.9 million, a rise of 1.7 million; in relative terms, the population in moderate poverty in the total changed from 34.9 to 35.4% in the same two periods. As a result of them, the poverty rate in Mexico increased from 41.9% in 2018 to 43.9% in 2020.

In addition, from the perspective of their cumulative change range, the moderate poverty and the extreme poverty have increased by 7.3 and 24.1%, respectively in 2 years; that is to say, extreme poverty has been up faster than the moderate poverty in terms of both the net increase and cumulative indicators. Therefore, with the increasing of poverty rate, the poverty problem in Mexico has also aggravated, that is, the poverty was more serious in 2020 than 2018.

#### Population in vulnerability

The data in Figure 3 also shows that over the past 2 years, the number of people living in vulnerability has decreased by 1.4 million, because of the growth of 2.7 million due to the deprivation of at least one of the 6 social rights in one hand; and in another, the reduction of 1.3 million due to their income lower than the poverty line.

#### Population neither in poverty nor in vulnerability

During the same period, the number of people who are neither in poverty nor in vulnerability has increased in the past 2 years, from 29.3 million to 29.8 million, with a net gain of 500,000 people.

From the above analysis, we can also find that the most fundamental reason for the reconfiguration of poverty situation in Mexico in the past 2 years is probably the decline of income amounts which led to double effects on poverty. On the one hand, the decrease of personal income caused the direct increase in the number of people whose income situated below the poverty line; on the other hand, it has also conducted to a rise of the residents tramped in the vulnerability. In addition, most of the residents who have been deprived the freedom in the access to health care security belong to the multidimensional poverty, that is, they are neither in vulnerability nor in the group that their personal income is higher than the poverty line and at the same time, can enjoy the freedom to develop their social rights fully.

**Figure 4:**
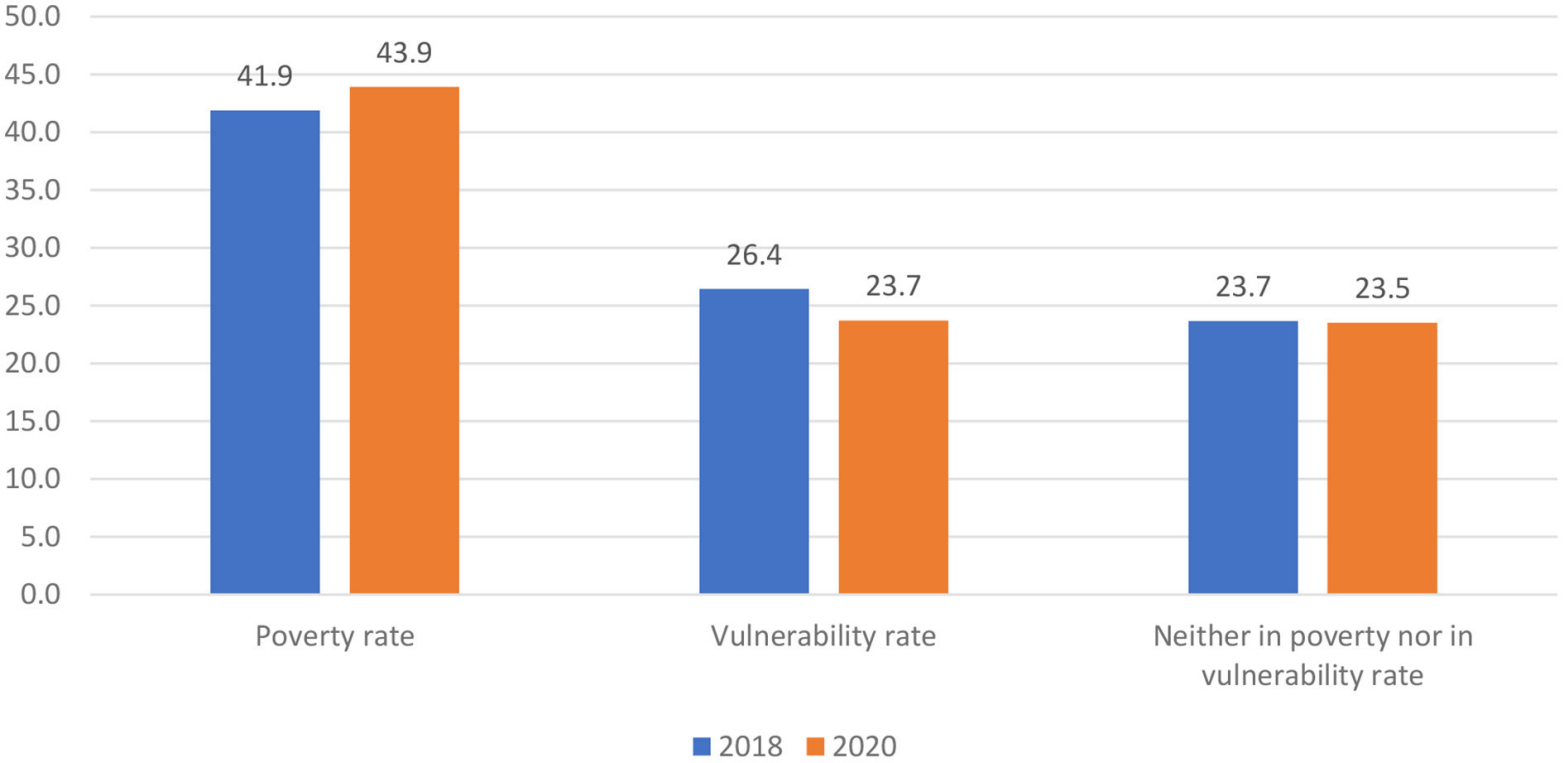
Changes in poverty structure of Mexican population, 2018–2020 (%). Source: Based on data published by the Mexican National Commission for the Evaluation of Social Development Policies.

Finally, although the absolute number of people who are neither in poverty nor in vulnerability has increased during 2018–2020, its growth rate is obviously lower than that of the total population of the whole country, which leads to a slightly decline of its proportion in the total population, from 23.7% in 2018 to 23.5% in 2020 (as shown in Figure 4). In other words, in the first 2 years of this administration, the participation of both the population in vulnerability and those neither in vulnerability nor in poverty in the national total has declined, and only the group in poverty has increased.

### The relationship between the change of Mexican poverty structure and COVID-19 pandemic

Considering the economic recession in 2019 and 2020 for two consecutive years, especially in 2020, in the face of sudden propagation of the COVID-19 pandemic and the consequently suspension of various economic activities, it is expected that the newly released poverty report should show more aggravated situation in poverty without any surprise. In fact, the Economic Commission for Latin America and the Caribbean (ECLAC) pointed out in the report ”Social Conditions in Latin America“ that the poverty rate in the region increased from 30.5% in 2019 to 33.7% in 2020, while the extreme poverty rate was 11.3 and 12.5%, respectively in the same 2 years, and the number of poor and extreme poor increased by 11.8 and 11.4%, respectively in 1 year (14). It can be seen that although the growth rate of poverty in Mexico is lower than the average level in Latin America, the number of extremely poor people in Mexico is twice the average level in the region.

It should be pointed out that among the 209 million people in poverty in Latin America in 2020, Mexico accounted for 26.7% of the total, which is higher than the proportion of the same country in the total population of the region in the precise period, which is about 19.0%. That is to say, in both relative and absolute terms the number of people in poverty under the influence of sudden propagation of the public sanitary emergencies, Mexico is more serious affected.

Additionally, the propagation of COVID-19 pandemic on poverty may extend its negative impacts on poverty to other senses not just only limited in the increase of poverty rate and the number of people in poverty. Firstly, for those workers inscribed in the local medical insurance systems, such as the El Instituto Mexicano del Seguro Social (IMSS), the El Instituto de Seguridad y Servicios Sociales para los Trabajadores del Estado (ISSSTE), etc., they can receive the health care services certainly but with different degrees of attentions due to their personal income amounts. It is common that those with higher personal income can get access to the health centers easier and receive the attention faster than the workers whose personal income is cataloged in the lower level. According to one analysis realized, in terms of both hospitalization rate and mortality caused by the COVID-19, the employees whose personal income is found in the bottom of deciles were more seriously affected in comparison to those located in the upper side of the deciles. Concretely, among the people with positive test results, those with lower incomes, in fact, had four times more the probability of being hospitalized than the richer; and in terms of mortality, those in the lowest decile had five times the probability to finishing dyed as those in the highest decile (15).

Secondly, in Mexico almost a half of the workers are found in the informal economic activities, and they hardly have the access to the health centers with free charge same as those employed in the formal sector, and these informal occupied people normally are cataloged in the bottom deciles of income. According to INEGI, in April of 2022, 55.5% of the occupied labor was cataloged as the informal workers (16). In this sense, their poverty situation may be even more severe in the face of the impact of COVID-19 pandemic.

### The impact of COVID-19 on the effect of poverty reduction policies

In the face of sudden sanitary emergencies, Mexico, like other countries, had experienced a long period of shutdown of economic activities. In the worst moment on April-May of 2020, the number of unemployed people in the whole country once exceeded 10 million, and the income of residents also dropped sharply. With respect to the economic growth, in 2020, after the economic recession in 2019, Mexican economy continued with another year down with a decreasing rate of 8.4% (calculated according to the seasonally adjusted constant price in 2013), which was not only one of the countries with the most serious economic decline in the G20, but also among the deepest ones in Latin America.

It can be said that COVID-19 pandemic is an unexpected event same as an uncontrollable factor that hit the economic growth and the poverty reduction programs and social relief measures applied by all of the authorities in its respective level. However, probably another element which cannot be depreciated should relate to government's public policies which could mitigate those negative effects on the economic growth and of course, on the increase of poverty rate and the number of people in poverty.

In fact, according to the data of ECLAC, in Latin America, the level of government transfer expenditure implemented by Mexico during the epidemic period was low, accounting for only 0.42% of GDP, which was significantly lower than that of Argentina (2.23%), Brazil (4.02%), Chile (1.83%), Colombia (1.16) and Peru (2.36%) in the same period (17). In other words, the economic growth rate could be less than the observed and the poverty situation could be more moderate if the public reactions would mitigate more the negative effects caused by the COVID-19.

**Figure 5:**
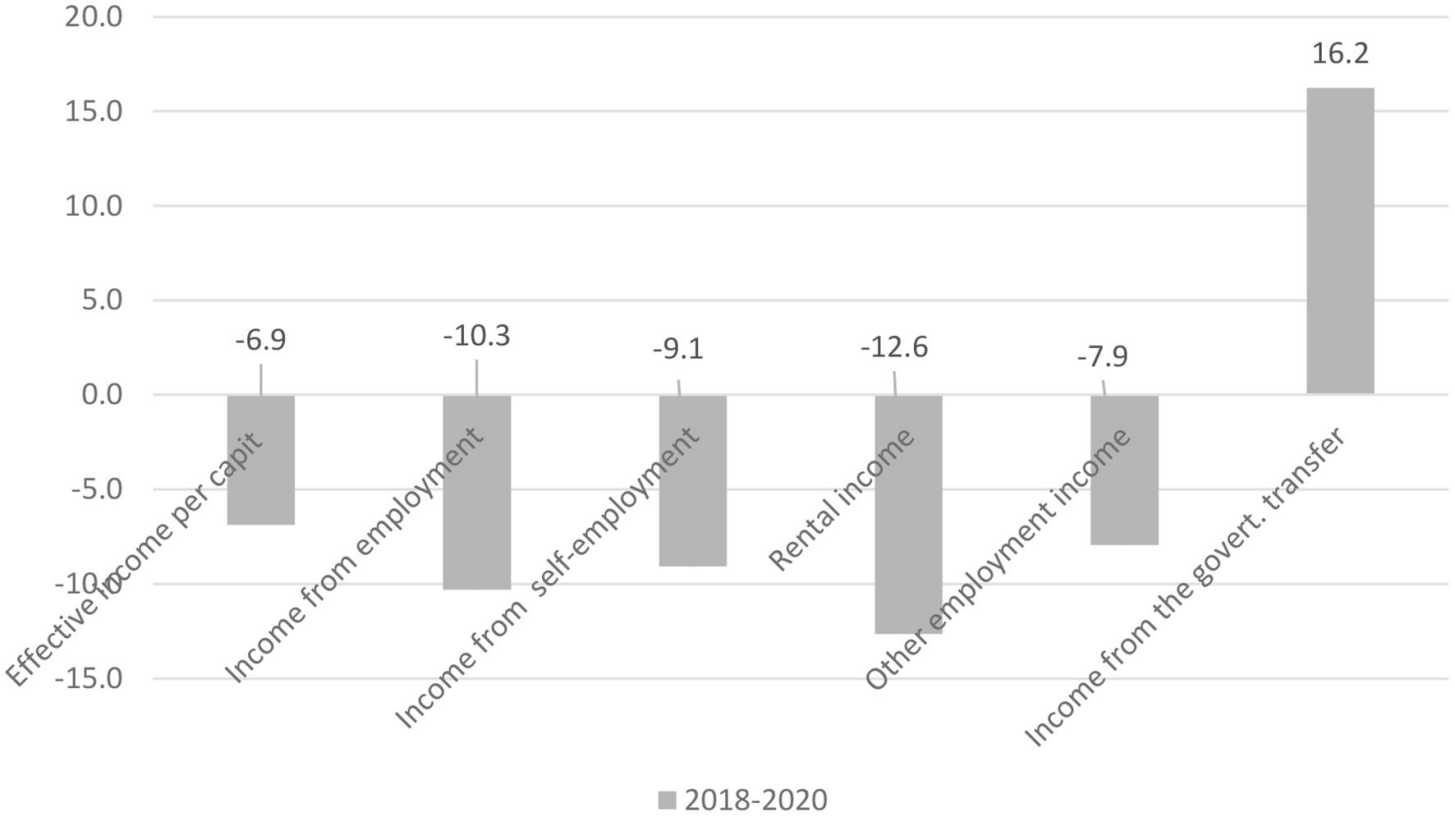
Change rate of per capita monthly cash income in Mexico (comparison of data in the same month of 2020 and 2018, %). Source: Calculation based on the data published by the INEGI.

Additionally, the data released by the CONEVAL showed that, compared with 2018, based on the constant prices in August 2020, the monthly income per capita in the whole country decreased by 6.9%, among the respective sources, the income from employed labor did by 10.3%, and from leased assets by 12.6% with the same direction. This situation not only reflects the sharp increase of unemployed people in that year, but also is related to the suspension of various economic activities during the epidemic. So during the 2 years from 2019 to 2020, although the government's transfer income has increased by 16.2%, due to formulation and implementation of the several social welfare policies, such as youth training programs and universal retirement subsidy programs for all elderly people, etc. they still cannot make up for the decrease in other aspects of income (as shown in Figure 5).

To a certain extent, it should not be belittled that all the programs destined to improve the social benefits taken by the current government thorough the increase the public expenditure have restrained the increase of poverty rate caused by economic recession, but the effect is not obvious. In 2018 (the last year of the previous government's administration), without the government's transfer income, the number of poor people would be 2.3 million more people in poverty; in 2020 (the second year of the current government's administration), there would be an amount of 2.5 million people were prevented from falling into poverty because of the government's increased income transfer. In this sense, the extraordinary growth of the public expenditure applied by this administration since 2019 only has avoided 200 thousand of people tramped in poverty.

In relative terms, it means an increase of 8.7% compared with the level observed in 2018. The reason is most likely related to the lack of focalization in the implementation of these policies, besides the negative effects generated by the COVID-19. In Mexico, a country serious affected by the inequality in the distribution of income with a wide gap between rich and poor, the wealth accumulated in the highest income decile is more than 20 times that of the lowest decile. During the last 40 years, the coefficient of Gini has increased from the end of 1980s to the middle of 1990s, and then showed a declining tendency until now (18, 19), however, Mexico still occupied as one of the most unequal countries all over the world and in 2020, the indicator was 0.450 (12).

At the same time, for the household cataloged in this decile, the government transfer normally represents the main source in their income, accounting for 47.0%; in the other extreme, for the households in the 10^th^ decile, the pension income is their main item, accounting for 70%, while government transfer income only accounts for 3.2%.

However, various policies with the purpose to alleviate the poverty for the households classified in the first decile have been applied uniformly among the people with abysmal difference of personal income amounts. The same quantity of government transfers but destined to the households in distinct decile will imply varied importance for their income, just as it showed before. It means that those in poverty really need the government's subsidies will receive the equal amounts than those classified in the highest decile of households, further aggravating or at least maintaining the existing unequal income distribution and reducing the efficiency in the reduction of poverty.

**Figure 6:**
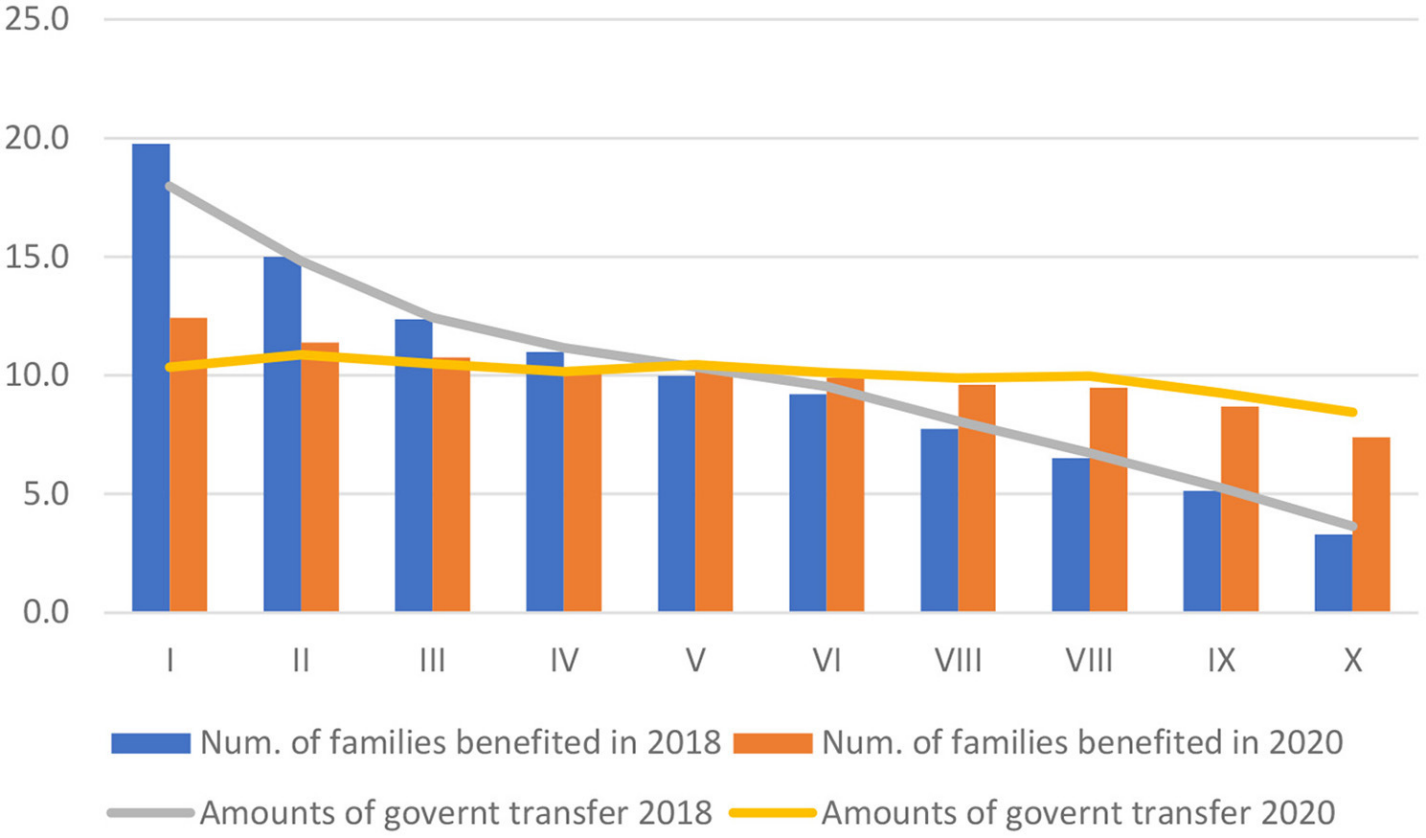
Structure of government transfer received by households according to income deciles in Mexico, 2018–2020 (%). Source: Calculation based on the data published by the INEGI.

The uniformity in the application of the respective programs based on the principal “Primero los pobres” to help the people in poverty has not generated the expected results in the alleviation of Mexico poverty at least in two aspects. Firstly, the government transfer has distributed equally to the population in different deciles of personal income which conducted the decrease of the household in the deciles I-IV, the families considered as the lover personal income, meanwhile the people in the highest deciles particularly in the X have been the most benefited. In fact, from 2018 to 2020, the number of families in the decile I reduced their participation in the total benefited by 7.3 percentage points from 19.8% in 2018 to 12.4% in 2020, in comparison to a net increase observed for those in the X decile by 4.1 percentage points from 3.3 to 7.4% in the same 2 years (Figure 6).

Secondly, in monetary terms, the total families classified in the X decile received 8.4% of all the governments transfer in 2020, that is, 4.8 percentage points more than that get in 2018 by 3.6%; in contrary, the households cataloged in the decile I registered a decline in the participation in the total governments transfer by 7.3 percentage points, from 18.0 to 10.3% during the same period. In other words, although the original intention of this government is to support the poor people, the actual implementation result is biased toward high-income families.

Therefore, the occurrence of COVID-19 pandemic has had a serious impact on the Mexican economy, resulting in a sharp decline in the local personal income level, which is undoubtedly the main reason for the increase in poverty rate. However, on the other hand, in the face of the adverse events, the shortage of focalization in the application of the respective programs dedicated to alleviate the poverty also played an important role in the redistribution of the benefits among the families classified in the deciles.

In related to satisfy the social rights demands, particularly in the health care services hit seriously by the COVID-19, the Mexican government has initiated the transformation of its social security systems by the consolidation of the public institutions such as the IMSS and ISSSTE, etc., and at the same time, by the assignation of more public expenditures in construction of new infrastructures and the improvement of the already existents.

## Conclusions

In order to promote the public social development policy and to guarantee the basic economic benefits and social rights for all of the families, particular those dropped in the poverty and margination, the National Commission for the Evaluation of Social Development Policies has specialized in the definition, identification and measurement of poverty and through the time has improved them.

At the same time, the reports regularly published by the same institution related to poverty every 2 years became an important basis for Mexico authorities in the respective level to do better in the formulation, implementation and monitoring the public social policies.

Although the current Mexican government has increased the public expenditure in the implementation public policies with the purpose to back up the families considered in poverty, holding the principle of taking care of the poor first, they can make up the losses caused by economic recession in face of the COVID-19. In this sense more people dropped in poverty since their personal income was lower than the poverty line, or they have been deprived at least one of the 6 social rights determined by the CONEVAL.

Additionally, the absence of focalization in the application of the respective programs dedicated to alleviate the poverty also played an important role in the redistribution of the benefits among the families classified in the deciles, and resulted in the reduction their efficiency in the reduction of poverty. Therefore, how to recover and improve the economic growth rate as soon as possible to overcome the negative effects generated by the COVID-19, is essential in the improvement of the households' living standard and the general social wellness, it should be accompanied at the same time by properly adjusting the actual poverty alleviation measures and social relief policies, focalized particularly in the low-income people.

At least until the 2^nd^ trimester of 2022, the Mexican economy has not yet recovered the lost in 2020, and the economic perspectives will be complicated with a lot of challenges, such as the increasing inflation pressure, interest rate hiking and the regional conflicts, etc., the poverty reduction in Mexico should be a long-term job and require the efforts not only to stimulate the economic growth but also to improve the income distribution (19).

## Data availability statement

The original contributions presented in the study are included in the article/supplementary material, further inquiries can be directed to the corresponding authors.

## Author contributions

All authors listed have made a substantial, direct, and intellectual contribution to the work and approved it for publication.

## Funding

This work was supported by the Project GBQY2022WT-69 sponsored by Ministry of Education of China, Project PAPIIT IN31102 (2020–2022) sponsored by National Autonomous University of Mexico.

## Conflict of interest

The authors declare that the research was conducted in the absence of any commercial or financial relationships that could be construed as a potential conflict of interest.

## Publisher's note

All claims expressed in this article are solely those of the authors and do not necessarily represent those of their affiliated organizations, or those of the publisher, the editors and the reviewers. Any product that may be evaluated in this article, or claim that may be made by its manufacturer, is not guaranteed or endorsed by the publisher.

## References

[1] Zongsheng C Yun H Yunbo Z. Application research and governance practice of multidimensional poverty theory and measurement method in China. Foreign Social Sciences. (2020) 15–34.

[2] López-Calva LF Ortiz-Juárez E. A vulnerability approach to the definition of the middle class. J Econ Inequal. (2014) 12:23–47. 10.1007/s10888-012-9240-5

[3] López-Calva LF Cruces G Lach S Ortiz-Juárez E. Clases medias y vulnerabilidad a la pobreza, reflexiones desde América Latina. El Trimestre Económico. (2014) 81:281–307. 10.20430/ete.v81i322.115

[4] Rowntree BS. Poverty: A Study of Town Life. London: Macmillan (1901).

[5] Kun Y Yifang L. Research on Anti-poverty Theory and Practice with Chinese Characteristics. Beijing: China Social Sciences Press (2016).

[6] Sen A. Viewing Development with Freedom. Beijing: Renmin University of China Press (2012).

[7] Consejo Nacional de Evaluación de la Política de Desarrollo Social. Metodología para la medición multidimensional de la pobreza en México (tercera edición). Ciudad de México: CONEVAL (2019). p. 68Ű71.

[8] Ministro de Desarrollo Social y Familia. Gobierno de Chile. Informe de desarrollo social 2018. Santiago de chile: MINDES (2018).

[9] Instituto Nacional de Estadística y Geografía. Cuantificación de la clase media en México 2010–2020. Ciudad de México: INEGI(2021).

[10] Presidencia de la República (2013) la Iniciativa de Decreto por el que se reforman adicionan y derogan diversas disposiciones de los Títulos Tercero Bisy Décimo Octavo de la Ley General de Salud. México. Available online at: http://www.diputados.gob.mx/PEF2014/ingresos/09_lpu_lsd.pdf

[11] Instituto Nacional de Estadística y Geografía. Encuesta Nacional de Ingresos y Gastos de los Hogares 2020. Ciudad de México: INEGI (2021).

[12] Consejo Nacional de Evaluación de la Política de Desarrollo Social. Medición multidimensional de la pobreza en México 2018–2020. Ciudad de México: CONEVAL (2021).

[13] Guerrero AL Méndez JSM. De Seguro Popular a INSABI Mayor población con menor atención [Z/OL].Available online at: https://ciep.mx/wp-content/uploads/2021/06/evolucion_insabi-2.pdf

[14] Comisión Económica para América Latina y el Caribe. Panorama Social de América Latina, Santiago: CEPAL (2021).

[15] Arceo-Gomez EO Campos-Vazquez RM. The income gradient in COVID-19 mortality and hospitalisation: An observational study with social security administrative records in Mexico. Lancet Reg Health Am. 10.1016/j.lana.2021.100115 34778865 PMC8578731

[16] INEGI. COMUNICADO DE PRENSA NÚM. 303/22, Indicadores de ocupación y empleo, abril de 2022 (2022). Available online at: https://www.inegi.org.mx/contenidos/saladeprensa/boletines/2022/iooe/iooe2022_05.pdf (accessed May 31, 2022).

[17] Comisión Económica para América Latina y el Caribe. Estudio Económico de América Latina y el Caribe 2020: principales condicionantes de las políticas fiscal y monetaria en la era pospandemia de COVID-19. Santiago: CEPAL (2021).

[18] Campos Vázquez RM Rodas Milian JA. Desigualdad en el ingreso: posibilidades de acción pública. Economía UNAM. (2019) 19:251–61.

[19] OECD. OECD Economic Surveys Mexico: Overview. OECD, Economics Department (2017). Available online at: https://www.oecd.org/economy/surveys/mexico-2017-OECD-Estudios-economicos-de-la-ocde-vision-general.pdf.p21

